# Uncovering the determinants of infant and toddler childcare demand in less-developed rural China: a machine learning perspective

**DOI:** 10.3389/fpubh.2026.1810627

**Published:** 2026-04-17

**Authors:** Tingting Fan, Jichang Guo

**Affiliations:** School of Education Science, Minzu Normal University of Xingyi, Xingyi, China

**Keywords:** childcare service quality, infant and toddler childcare demand, less-developed rural China, machine learning, random forest

## Abstract

**Introduction:**

China faces a severe imbalance between the supply and demand of formal childcare services for infants and toddlers aged 0–3, with rural research and resource allocation falling far behind urban areas.

**Objective:**

This study aimed to identify key determinants of rural childcare demand in western China’s less-developed regions and to screen the optimal predictive machine learning (ML) prediction model, based on Andersen’s Behavioral Model.

**Methods:**

A cross-sectional survey with purposive and multi-stage sampling was conducted in southwestern Guizhou Province, collecting valid data from 1,116 rural families with infants and toddlers. Seven ML algorithms were applied to construct childcare demand prediction models with comprehensive model evaluation, SHAP analysis for feature identification, and threshold analysis for key factors performed subsequently.

**Results:**

The random forest (RF) model was identified as the optimal model, demonstrating robust generalization and discriminative ability. SHAP analysis revealed childcare flexibility, overall childcare quality, early education, and teacher professionalism as the four core positive determinants of rural childcare demand. Threshold analysis further defined the optimal critical values of these key factors and verified their strong practical predictive performance, with childcare flexibility and overall quality showing the most prominent effects.

**Conclusion:**

This study confirms that machine learning can effectively identify the determinants of rural childcare demand. The four service-related factors are the most influential drivers. The findings provide empirical evidence for optimizing rural childcare services and formulating demand-oriented childcare policies to promote the high-quality and inclusive development of rural childcare systems in less-developed regions in China.

## 1 Introduction

In recent years, the demand for childcare services among families with infants and young children in China has become increasingly prominent. Childcare services are not only a critical policy approach to improve fertility rates but also an important measure to promote social welfare. In China, formal childcare services are mainly provided by professional institutions for children under three years old (1). These services are defined as universal, purposeful, and structured care, guidance, and developmental support for infants and toddlers outside the family, aiming to promote the physical and mental development of children aged 0–3 years and provide evidence-based parenting guidance for caregivers (2). This definition indicates that childcare goes beyond basic supervision and integrates core elements of early education and holistic child development.

Despite strong demand for childcare among families with children under 3, overall supply remains seriously insufficient. In China, 68.4% of families report a need for childcare, yet the enrollment rate for children aged 0–3 years is only 5.5% (3). A study showed that 52.7% of parents prefer formal childcare services in Chongqing, China (4). In Hong Kong, demand is particularly high among dual-working parents, but limited nursery places and long waiting lists remain major constraints (5). In Europe, formal childcare enrollment for this age group varies widely, from less than 10% in Poland to over 40% in Portugal (6). Historically, childcare services have been underprioritized, resulting in weak infrastructure and ongoing challenges in accessibility, quality, and governance (3). Services for infants and toddlers still suffer from supply–demand imbalances, uneven resource distribution, and insufficient supervision (7). Therefore, a thorough analysis of factors influencing parental childcare demand is essential to improve the quality and accessibility of childcare provision (8).

Demand for formal childcare among children aged 0–3 years is driven by a complex set of factors. First, socioeconomic status plays a key role. Some studies indicated that higher household income and parental education are associated with stronger preferences for formal childcare (4, 9, 10). Urban families generally exhibit higher demand for formal childcare and greater willingness to pay for such services (1, 4). Second, family care arrangements significantly shape childcare demand. Families with multiple children also demonstrate higher demand for such services (11, 12). In contrast, the widespread use of grandparental or other informal family care can substantially reduce parental reliance on formal childcare services (4). Third, service quality strongly influences parental childcare decisions. Parents prioritize institutional safety, professional staffing, appropriate teacher-child ratios, and comprehensive services that cover nutritional support, self-care training, and cognitive development (1, 5). Trust in childcare institutions, together with awareness of available services and relevant policies, is positively associated with childcare utilization (12), and caregiver professionalism is regarded as central to overall service quality (8). In addition, convenience and geographic accessibility represent key considerations in parental choice (13). A study in Florida further documented substantial spatial mismatch, with families traveling considerably farther than necessary to access care, a pattern that disproportionately affects families with infants (14).

Taken together, existing evidence highlights urban families as a group with substantial childcare demand, yet research focusing on the needs and associated determinants among rural families remains relatively limited. Existing studies focus heavily on urban populations and pay insufficient attention to rural and county-level regions (8). Childcare resources in rural areas are far scarcer than in urban areas (15). Rural residents show lower childcare utilization, which can be explained by lower socioeconomic status, higher costs relative to informal care, and poor service coverage and accessibility (1, 16). Employment types for rural women, such as agricultural work and local vending, often allow them to combine work and childcare (17). In addition, traditional family care supported by grandparents remains common in rural China (3), and rural households typically benefit from stronger social support and more extensive extended family networks (18). Existing research on rural childcare demand has primarily focused on household economic conditions, occupational flexibility, and intergenerational support. However, current frameworks seldom incorporate service quality attributes including institutional environment, care standards, and educational content as key determinants of demand and willingness to pay.

Therefore, this study is theoretically grounded in Andersen’s Behavioral Model of Health Services Use (19), a classic analytical framework for interpreting individuals’ health service utilization behavior and equitable access. According to its core theoretical logic, the demand for health services is determined by the extent to which individuals with specific predisposing characteristics utilize medical services under the constraints of personal and community resources (20). The model has undergone several iterations, yet its core theoretical framework, the logical relationships among Predisposing Characteristics, Enabling Resources, Need, and Use, has remained stable (19). This provides the theoretical basis for this study to establish associations between variables and childcare needs. Among these, predisposing characteristics are the fundamental factors influencing service demand, while enabling resources constitute the key supporting conditions for the transformation of demand intention into actual demand. The two jointly affect the formation and decision-making of demand. This core logic provides clear theoretical guidance for this study to integrate the three variable dimensions of maternal, family, and childcare services and establish their associations with childcare needs.

Compared with traditional statistical methods such as logistic regression, which rely on linear assumptions and predefined theoretical frameworks, machine learning (ML) approaches offer clear advantages in exploring childcare demand. First, ML effectively captures nonlinear relationships and complex interactions among multidimensional factors, which is essential for understanding demand shaped by combined social, family, and service-related conditions (21–23). Second, tree-based and ensemble methods such as random forest (RF) and XGboost provide strong predictive performance and reliable feature ranking from high-dimensional data, performing better than conventional methods in identifying key determinants (24). Third, interpretable techniques such as SHAP (SHapley Additive exPlanations) values help overcome the black-box problem and enable clear interpretation while maintaining high accuracy (23–25). These advantages make ML highly suitable for exploring patterns in rural childcare demand, where traditional analytical frameworks may be insufficient.

Based on existing research gaps and the strengths of ML, this study has two main objectives: (a) To evaluate and compare the predictive performance of seven ML algorithms, including logistic regression (LR), decision tree (DT), RF, Adaboost, support vector machine (SVM), XGboost, and lightGBM, in identifying factors associated with childcare demand among rural families with infants and toddlers; (b) To leverage the optimal model combined with SHAP analysis to determine the main factors affecting the childcare demand of rural families, quantify the relative importance of these key influencing factors, and clarify the underlying mechanisms of childcare demand in rural households; (c) To conduct threshold analysis on the optimal model and key influencing factors, determine the critical values of variables affecting the childcare demand of rural families, and provide practical reference.

## 2 Methods

### 2.1 Participants

A cross-sectional survey was conducted in December 2025 across multiple districts in southwestern Guizhou Province, China. As a less-developed western region, Guizhou has a rural population accounting for nearly half its total residents, a proportion significantly higher than the national average. Widespread rural–urban labor migration strongly shapes childcare demand among families with infants and toddlers. Southwestern Guizhou provides a typical and representative setting for examining childcare needs among rural Chinese families with young children.

The study adopted a combined purposive and multi-stage sampling design. Participants were rural families with infants and young children from eight counties or county-level cities in southwestern Guizhou. Five administrative villages were randomly selected from each county as primary sampling units. Eligible families were recruited through village-level authorities, with the inclusion criterion of having an expected child or a child aged under 3 years. Written informed consent was obtained from all participants prior to data collection. Given that mothers usually serve as primary caregivers in rural Guizhou households, all questionnaires were completed by the child’s mother to ensure data reflected key caregiver perceptions.

Overall, 1,200 questionnaires were distributed, with 150 questionnaires per county or county-level city. Surveys were administered to eligible participants by local staff responsible for women’s and children’s affairs. After data quality control, 1,116 valid and complete responses were finally obtained for subsequent analysis. Participant characteristics are presented in Table 1.

**Table 1:** Characteristics of participants.

Variables/features		*n*	Childcare demand		*χ*^2^ or *t*	*p*
			No demand (*n* = 422)	Demand (*n* = 694)		
Maternal age	1 = 2000s	150	67 (44.67)	83 (55.33)	5.802	0.122
	2 = 1990s	752	285 (37.90)	467 (62.10)		
	3 = 1980s	202	65 (32.18)	137 (67.82)		
	4 = 1970s	12	5 (41.67)	7 (58.33)		
Maternal educational level	1 = Primary school	67	26 (38.81)	41 (61.19)	2.811	0.590
	2 = Middle school	352	143 (40.63)	209 (59.37)		
	3 = High school or technical secondary school	164	58 (35.37)	106 (64.63)		
	4 = Junior college	180	61 (33.89)	119 (66.11)		
	5 = Bachelor or higher	353	134 (37.96)	219 (62.04)		
Number of children born	1 = One child	309	127 (41.10)	182 (58.90)	2.010	0.366
	2 = Two children	557	205 (36.80)	352 (63.20)		
	3 = Three or more children	250	90 (36.00)	160 (64.00)		
Number of grandparental childcare	1 = 0 people	284	95 (33.45)	189 (66.55)	11.289	0.010
	2 = 1 people	371	145 (39.08)	226 (60.92)		
	3 = 2 people	365	132 (36.16)	233 (63.84)		
	4 = 3 people or more	96	50 (52.08)	46 (47.92)		
Monthly household income	1 = Less than 4000RMB	426	190 (44.60)	236 (55.40)	15.600	0.004
	2 = 4,000–6000RMB	357	113 (31.65)	244 (68.35)		
	3 = 6,001–8000RMB	160	61 (38.13)	99 (61.87)		
	4 = 8,001–10000RMB	84	27 (32.14)	57 (67.86)		
	5 = More than 10000RMB	89	31 (34.83)	58 (65.17)		
Childcare fees	1 = More than 2000RMB	58	17 (29.30)	41 (70.70)	16.038	0.003
	2 = 1,501–2000RMB	202	60 (29.70)	142 (70.30)		
	3 = 1,001–1500RMB	174	76 (43.70)	98 (56.30)		
	4 = 501–1000RMB	331	116 (35.00)	215 (65.00)		
	5 = Less than 500RMB	351	153 (43.60)	198 (56.40)		
Distance from home	1 = Beyond 5 km	58	27 (46.55)	31 (53.45)	5.376	0.251
	2 = Within 5 km	217	77 (35.48)	140 (64.52)		
	3 = Within 3 km	318	112 (35.22)	206 (64.78)		
	4 = Within 1 km	349	131 (37.54)	218 (62.46)		
	5 = Within 0.5 km	174	75 (43.10)	99 (56.90)		
Childcare flexibility		1,116	4.44 (2.09)	6.21 (1.21)	−15.855	<0.001
Overall childcare quality		1,116	4.63 (2.16)	6.27 (1.12)	−14.522	<0.001
Environmental facilities		1,116	5.18 (2.06)	6.17 (1.14)	−9.037	<0.001
Health and hygiene		1,116	5.53 (2.15)	6.54 (0.98)	−9.068	<0.001
Nursing care		1,116	5.55 (2.13)	6.56 (0.92)	−9.233	<0.001
Early education		1,116	5.31 (2.14)	6.50 (0.96)	−10.838	<0.001
Teacher professionalism		1,116	5.35 (2.15)	6.54 (0.95)	−10.783	<0.001

Values are presented as mean (standard deviation) [M (SD)] for childcare flexibility, overall childcare quality, environmental facilities, health and hygiene, nursing care, early education, and teacher professionalism. All other variables are reported as frequencies and percentages (*n*, %).

### 2.2 Measures

This study examined the factors influencing childcare demand among rural families with infants and toddlers in Guizhou Province, focusing on three key dimensions: maternal factors, family factors, and childcare service factors.

#### 2.2.1 Outcome variable

The primary outcome variable was childcare demand, which was dichotomously coded as 0 (no demand) and 1 (demand). The question is “If childcare services could meet your needs, would you send your child under the age of 3 to a childcare facility?” Parents need to respond as follows: Yes indicates a demand, and No indicates no demand. In existing literature on childcare needs research, single-item dichotomous questions are commonly used for measurement (7, 12). The present study adopts the same approach. Furthermore, this study aims to investigate the influencing factors of childcare demand, as classifying childcare demand into more than three categories would complicate the machine learning model.

#### 2.2.2 Maternal factors

The predisposing characteristics in the Andersen’s model refer to individual demographic attributes, including age, gender, educational level, and so on.

Two key explanatory variables were included: *maternal age* (categorized into four cohorts: 1970s, 1980s, 1990s, and 2000s), and *maternal educational level* (five categories ranging from primary school to bachelor or higher).

Notably, this study does not include maternal employment status in the analysis. This is mainly because preliminary field investigations revealed that the core responsibilities of mothers of infants and young children in rural areas of the study region are primarily family care and child-rearing. They generally plan to re-enter the labor market only after their children reach a certain age. Given the homogeneous employment status, its impact on childcare demand is not significant, so it was not included in the research variable system.

#### 2.2.3 Family factors

In the Andersen model, enabling resources refer to various conditions for the transformation of needs, including internal family resources such as economic status and family support networks. Three explanatory variables were considered in this dimension:

*Number of children born* is classified into three ordinal categories: 1 child, 2 children, and 3 or more children. *Number of grandparental childcare* is categorized into four groups: no support, support from 1 people, support from 2 people, and support from 3 or more people, which aligns with China’s long-standing tradition of intergenerational care. *Monthly household income* is stratified into five groups (<4,000 RMB, 4,000–6,000 RMB, 6,001–8,000 RMB, 8,001–10,000 RMB, and >10,000 RMB).

#### 2.2.4 Childcare service factors

In the Andersen’s model, enabling resources include not only internal family resources but also external social resources such as the provision of community services. The external social resources focuses on the supply-side characteristics of childcare services. Drawing on the four-dimensional framework of childcare choice (26). In light of the current supply status of childcare services in the study area, targeted adjustments and refinements are made to social resource variables.

*Availability* refers to the degree of alignment between childcare services and family needs in terms of supply quantity, service hours, and appropriate age groups. The preliminary investigation shows that formal childcare institutions have not yet been established in rural areas of the study region, and parents have reached a consensus on the appropriate age for infants and young children to attend childcare. As a result, differences in supply quantity and appropriate age have no significant impact on childcare demand. Therefore, the flexibility of childcare services is selected as the core predictor variable to reflect the degree of matching between service hours and family care needs. *Childcare flexibility* measures the extent to which childcare institutions’ operating arrangements meet rural families’ demand. The question is “How much influence does the flexibility of childcare services (e.g., full-day, half-day, hourly, holiday care) have on your choice of a childcare institution?” It is rated on a 7-point Likert scale (1 = no influence at all, 7 = very strong influence).

*Accessibility* refers to the accessibility of childcare services in terms of geographical location and service information. Preliminary research indicates that parents of infants and young children in the study area have limited channels to obtain childcare information, and the impact of information accessibility on childcare demand is not significant. Therefore, only the distance from childcare institutions to home is selected as a predictor variable to measure the physical spatial accessibility of services. *Distance from home* to childcare institutions is divided into five categories (≤0.5 km, 0.5–1 km, 1–3 km, 3–5 km, and >5 km), where higher scores indicated closer proximity, to explore the impact of commuting distance on rural families’ childcare demand.

*Affordability* refers to the economic capacity of families to bear childcare expenses. The monthly fee standard of childcare institutions is selected as the core predictor variable, which directly reflects families’ willingness and ability to pay for childcare services. *Childcare fees* are stratified into five groups (<500 RMB, 500–1,000 RMB, 1,001–1,500 RMB, 1,501–2,000 RMB, and >2,000 RMB per month) based on the monthly income levels of rural households in Guizhou, with lower fees assigned higher scores.

*Quality* refers to the comprehensive level of services provided by childcare institutions. This study employs six items to investigate the influence of childcare service quality on childcare demand. Each of these aspects is assessed using one question. *Overall childcare quality* reflects the impact of the comprehensive service quality of childcare institutions on rural families’ choice of childcare institutions. The question is “To what extent does standardized, high-quality childcare service influence your choice of a childcare institution?” *Environmental facilities* focuses on the impact of the physical environment and facilities on rural families’ choice of childcare institutions. The question is “To what extent does the safe environment and facilities as well as appropriate toys and teaching aids in childcare institutions influence your choice of such institutions?” *Health and hygiene* investigates the role of sanitation practices in rural families’ decision-making regarding childcare institutions. The question is “To what extent does the standardized and effective implementation of daily hygiene, disinfection, health checks, and disease prevention and control in childcare institutions influence your choice of such institutions?” *Nursing care* explores how evidence-based care for infants and young children affect rural families’ choice of childcare institutions. The question is “To what extent does the scientific care of children’s diet, sleep, toilet and other daily routines influence your choice of a childcare institution?” *Early education* assesses the impact of developmentally appropriate early education content on rural families’ selection of childcare institutions. The question is “To what extent does the provision of age-appropriate early childhood education activities that promote children’s all-round development influence your choice of a childcare institution?” *Teacher professionalism* analyzes how the professional competence of childcare teachers influences rural families’ choice of childcare institutions. The question is “To what extent do teachers’ professional qualifications and caring attitude influence your choice of a childcare institution?” Some studies suggest that the seven-point Likert scale closely resembles continuous scale characteristics and enhances the reliability and validity of questionnaires (27, 28). Therefore, all the above variables are rated on a 7-point Likert scale (1 = no influence at all, 7 = very strong influence).

### 2.3 Statistical analysis

A series of systematic statistical analyses and machine learning model constructions were conducted to explore the characteristics of infant childcare demand and establish effective predictive models.

#### 2.3.1 Descriptive and comparative analysis

The total size of the classified dataset was 1,116, including 422 subjects in the No Demand group and 694 subjects in the Demand group. Descriptive statistical analysis was performed using SPSS 26.0 software to summarize the demographic, socioeconomic, and childcare-related characteristics of these study subjects. Appropriate statistical tests were adopted to compare the differences in these variables between the two groups, with independent t-tests used for continuous variables and chi-square tests applied for categorical variables.

#### 2.3.2 Construction and hyperparameter optimization of ML models

Seven machine learning algorithms were employed to construct predictive models for infant childcare demand, including LR, DT, RF, Adaboost, SVM, XGBoost, and LightGBM.

Before model training, the entire dataset was randomly shuffled to eliminate the impact of sample order on model training. Stratified sampling was then implemented to ensure the consistent distribution of the two groups in the training set and test set, with 20% of samples extracted from each group to form the test set and the remaining 80% serving as the training set. For each of the seven algorithms, 5-fold cross-validation combined with grid search was used to optimize hyperparameters. The training set was divided into 5 mutually exclusive subsets of equal size, and in each iteration 4 subsets were used as the training subset and 1 subset as the validation subset. This process was repeated 5 times to ensure that each subset was used as the validation subset once. Grid search was applied to traverse all combinations of hyperparameter candidates, and the combination with the best performance on the validation subset was selected as the optimal hyperparameter configuration for the corresponding algorithm to avoid model overfitting and improve model generalization ability.

#### 2.3.3 Handling of imbalanced data

Notably, the dataset exhibited an imbalanced distribution between the two groups, with a clear disparity in sample sizes between the No Demand group (422 subjects) and the Demand group (694 subjects). Such data imbalance may lead to bias in model training, favoring the majority class and reducing the predictive accuracy for the minority class. To address this issue and improve the reliability and generalization ability of the predictive models, three common imbalanced data processing methods were employed (29, 30).

SMOTE (Synthetic Minority Oversampling Technique) generates synthetic samples of the minority class by interpolating between existing minority class samples, thereby balancing the sample size between the minority and majority classes without losing key information of the minority class. ADASYN (Adaptive Synthetic Sampling), an improved version of SMOTE, adaptively adjusts the sampling rate according to the density of minority class samples by generating more synthetic samples for minority samples that are difficult to learn, which effectively alleviates the problem of over-sampling noise caused by SMOTE. RandomUnderSampler reduces the sample size of the majority class by randomly selecting a subset of the majority class samples to make the sample sizes of the two groups approximately equal, and it avoids excessive loss of majority class information by retaining representative samples.

#### 2.3.4 Comprehensive evaluation of model performance

After hyperparameter optimization, all models built on the original dataset and the three imbalanced processed datasets were evaluated on the test set using a comprehensive set of evaluation metrics to quantify their predictive capacity and comparative performance. These metrics included the Receiver Operating Characteristic (ROC) curve, Area Under the Curve (AUC), sensitivity, specificity, accuracy, precision, and F1-score, and an AUC value closer to 1 indicates a superior discriminative ability of the model (31, 32).

Additional analyses were conducted to further comprehensively evaluate the models. To assess the calibration ability of the optimal model, which refers to the consistency between the predicted probability of childcare demand and the actual occurrence probability, Sigmoid calibration (also known as Platt scaling) was adopted to calibrate the model. A calibration curve was plotted to visually reflect the calibration effect, and the Brier score was calculated to quantitatively evaluate the calibration accuracy with lower Brier scores indicating better calibration performance.

Decision curve analysis and precision-recall analysis (21, 31, 33, 34) were employed to further assess the practical application value and predictive reliability of the optimal model, complementing the comprehensive evaluation of model performance and providing actionable insights for the formulation of rural childcare policies. The Mann–Whitney U (35–37) test was performed to conduct pairwise comparisons of the prediction results among the seven models, aiming to verify the statistical significance of differences in performance between each pair of models. Additionally, the Youden J index was used to conduct threshold analysis on the optimal model and key predictive indicators to determine the optimal cut-off value for predicting infant childcare demand and improve the practical operability of the model (33, 34).

#### 2.3.5 Correlation analysis and multicollinearity test

To explore the correlation among the predictive variables and avoid the impact of multicollinearity on model performance, a correlation heatmap was plotted to visually display the correlation coefficients between variables. A multicollinearity test was also conducted with the variance inflation factor (VIF) as the evaluation index, and a VIF value less than 10 was considered to indicate no significant multicollinearity, ensuring the rationality and independence of the variables used in model construction.

## 3 Results

### 3.1 Characteristics of participants

Of the 1,200 distributed questionnaires, 1,116 were valid (response rate: 93%). Most mothers were post-1990s (67.38%, *n* = 752). Rural mothers had low educational attainment (37.54% middle school or below, *n* = 419). 65.95% (*n* = 736) of families received 1–2 grandparents’ childcare support, and 38.17% (*n* = 426) had monthly household income <4,000 RMB. Other details are presented in Table 1.

### 3.2 Machine learning model construction and performance comparison

#### 3.2.1 Machine learning model construction and the selection of the optimal model

In this study, all variables were used to construct predictive models for childcare demand using seven ML algorithms, including four tree-based models (RF, lightGBM, XGboost, DT) and three traditional models (Adaboost, SVM, LR). The optimal hyperparameters selected for each algorithm are listed in Table 2.

**Table 2:** The optimal hyperparameters for each algorithm.

Models	Optimized hyperparameters
LR	C: 1; max_iter: 1000; penalty: l2; solver: liblinear
DT	criterion: gini; max_depth: 10; min_samples_leaf: 4; min_samples_split: 10
RF	max_depth: None; max_features: sqrt; min_samples_leaf: 1; min_samples_split: 2; n_estimators: 50
Adaboost	algorithm: SAMME; learning_rate: 1.0; n_estimators: 200
SVM	C: 10; gamma: 0.1; kernel: rbf; probability: True
XGboost	colsample_bytree: 0.8; learning_rate: 0.1; max_depth: 7; min_child_weight: 1 n_estimators: 50; subsample: 0.8
LightGBM	learning_rate: 0.2; max_depth: −1; min_child_samples: 10; n_estimators: 200; num_leaves: 100; subsample: 0.8; verbose: −1

All hyperparameters were optimized via grid search combined with 5-fold cross-validation to mitigate overfitting and improve model generalization. Parameters are presented as “parameter: value” and separated by semicolons for conciseness.

The performance of seven models was assessed on the training and independent test sets using AUC, accuracy, sensitivity, specificity, precision, and F1-score, with detailed data presented in Table 3 (training set) and Table 4 (test set).

**Table 3:** Performance evaluation of seven ML models on the training set (M ± SE).

Model	AUC	Accuracy	Sensitivity	Specificity	Precision	F1
RF	1.000 ± 0.001	0.997 ± 0.004	1.000 ± 0.000	0.991 ± 0.006	0.995 ± 0.005	0.997 ± 0.003
LightGBM	1.000 ± 0.000	0.997 ± 0.004	0.998 ± 0.003	0.994 ± 0.005	0.996 ± 0.004	0.997 ± 0.003
SVM	0.980 ± 0.009	0.939 ± 0.016	0.986 ± 0.008	0.864 ± 0.023	0.922 ± 0.018	0.953 ± 0.014
XGBoost	0.978 ± 0.010	0.926 ± 0.017	0.986 ± 0.008	0.828 ± 0.025	0.904 ± 0.019	0.943 ± 0.015
DT	0.927 ± 0.017	0.853 ± 0.023	0.932 ± 0.017	0.724 ± 0.029	0.848 ± 0.024	0.888 ± 0.021
AdaBoost	0.859 ± 0.023	0.789 ± 0.027	0.919 ± 0.018	0.576 ± 0.032	0.781 ± 0.027	0.844 ± 0.024
LR	0.817 ± 0.025	0.759 ± 0.028	0.870 ± 0.022	0.576 ± 0.032	0.772 ± 0.028	0.818 ± 0.025

**Table 4:** Performance evaluation of seven ML models on the test set (M ± SE).

Model	AUC	Accuracy	Sensitivity	Specificity	Precision	F1
RF	0.861 ± 0.045	0.817 ± 0.051	0.878 ± 0.043	0.718 ± 0.059	0.836 ± 0.049	0.856 ± 0.046
LightGBM	0.858 ± 0.046	0.799 ± 0.052	0.842 ± 0.048	0.729 ± 0.058	0.836 ± 0.049	0.839 ± 0.048
SVM	0.827 ± 0.050	0.799 ± 0.052	0.878 ± 0.043	0.671 ± 0.062	0.813 ± 0.051	0.844 ± 0.047
XGBoost	0.789 ± 0.053	0.781 ± 0.054	0.885 ± 0.042	0.612 ± 0.064	0.788 ± 0.053	0.834 ± 0.049
DT	0.765 ± 0.056	0.723 ± 0.059	0.827 ± 0.049	0.553 ± 0.065	0.752 ± 0.057	0.788 ± 0.054
AdaBoost	0.715 ± 0.059	0.710 ± 0.059	0.849 ± 0.047	0.482 ± 0.065	0.728 ± 0.058	0.784 ± 0.054
LR	0.707 ± 0.060	0.670 ± 0.062	0.755 ± 0.056	0.529 ± 0.065	0.724 ± 0.059	0.739 ± 0.057

Tree-based models outperformed traditional models significantly on the training set, with prominent advantages in F1-score. RF and lightGBM delivered the best performance, both achieving a near-perfect AUC of 1.000 (1.000 ± 0.001 and 1.000 ± 0.000, respectively), an identical accuracy of 0.997 ± 0.004, and highly comparable F1-scores (0.997 ± 0.003 for both). RF reached a perfect sensitivity of 1.000 ± 0.000, while lightGBM had a slightly higher specificity of 0.994 ± 0.005; both maintained a precision above 0.995. XGboost (AUC: 0.978 ± 0.010, F1-score: 0.943 ± 0.015) and DT (AUC: 0.927 ± 0.017, F1-score: 0.888 ± 0.021) showed gradual performance decline but still retained strong classification capacity, with XGboost’s sensitivity (0.986 ± 0.008) comparable to RF and lightGBM. By contrast, traditional models exhibited notably poorer training performance: LR performed the worst with the lowest AUC (0.817 ± 0.025) and F1-score (0.818 ± 0.025); Adaboost had the lowest accuracy (0.789 ± 0.027), and both Adaboost and LR showed a low specificity of 0.576 ± 0.032. Though SVM performed the best among traditional models (AUC: 0.980 ± 0.009, F1-score: 0.953 ± 0.014), it was still inferior to top tree-based models and had an unsatisfactory specificity (0.864 ± 0.023).

All models exhibited a moderate performance decline on the independent test set, consistent with typical machine learning generalization trends, yet tree-based models remained dominant, with RF identified as the optimal model. RF outperformed all counterparts across all six metrics, achieving the highest AUC (0.861 ± 0.045), accuracy (0.817 ± 0.051), sensitivity (0.878 ± 0.043), precision (0.836 ± 0.049), and F1-score (0.856 ± 0.046), with a well-balanced performance despite a slightly lower specificity (0.718 ± 0.059) than lightGBM. LightGBM followed closely (AUC: 0.858 ± 0.046), with the highest specificity (0.729 ± 0.058) among tree-based models and a high sensitivity of 0.842 ± 0.048, but slightly lower accuracy (0.799 ± 0.052) and F1-score (0.839 ± 0.048) than RF. Traditional models suffered further performance degradation on the test set. Adaboost performed the worst, with the lowest AUC (0.715 ± 0.059) and specificity (0.482 ± 0.065) across all models. LR had the lowest accuracy (0.670 ± 0.062) and F1-score (0.739 ± 0.057) among traditional models, while SVM also showed low specificity (0.671 ± 0.062), reflecting far inferior generalization ability compared to tree-based models.

**Figure 1:**
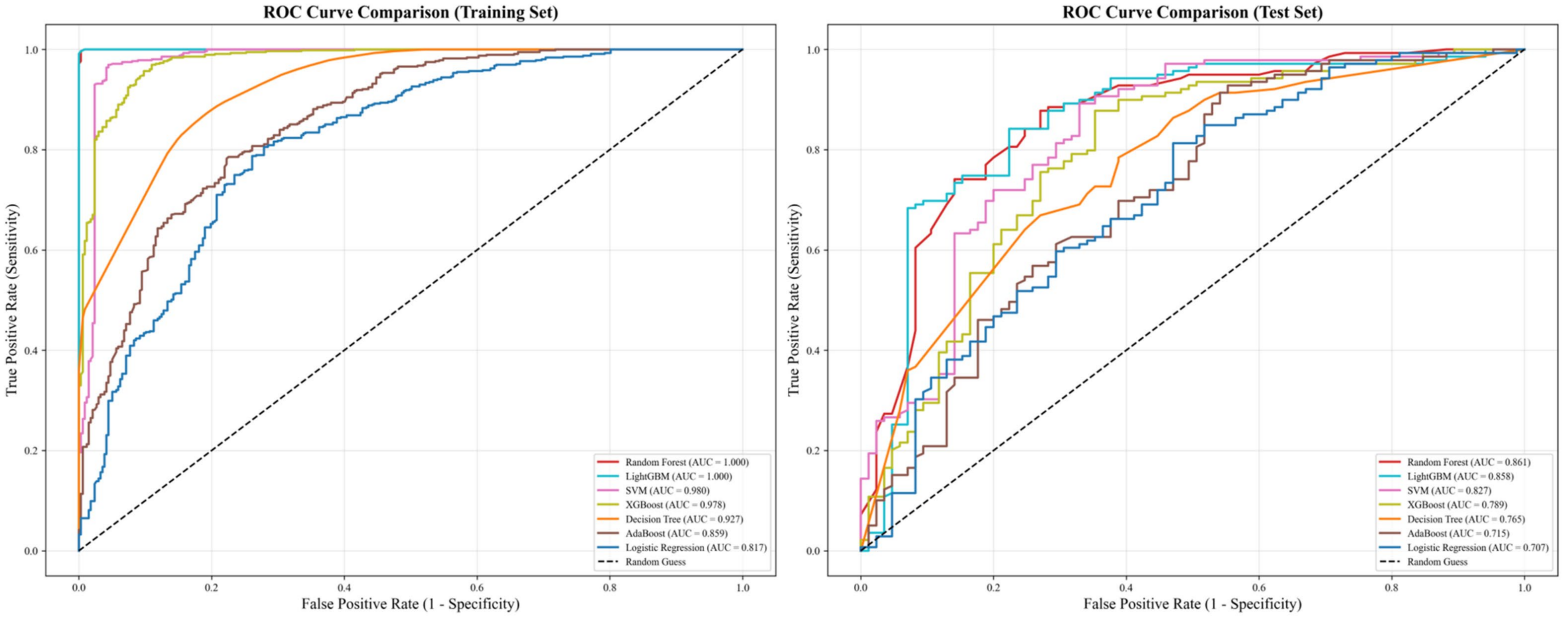
ROC curve comparison of training set and test set across seven ML algorithms.

In summary, tree-based models outperformed traditional models across all metrics on both datasets, demonstrating excellent training performance and robust generalization. RF was the optimal model for this classification task: it achieved top-tier performance on the training set, maintained the highest scores across all key metrics on the test set, and exhibited a relatively small performance decline with a well-balanced ability to identify the samples with childcare demand and exclude the samples without childcare demand, thus showing strong practical utility. The ROC curves of all models are shown in Figure 1.

**Figure 2:**
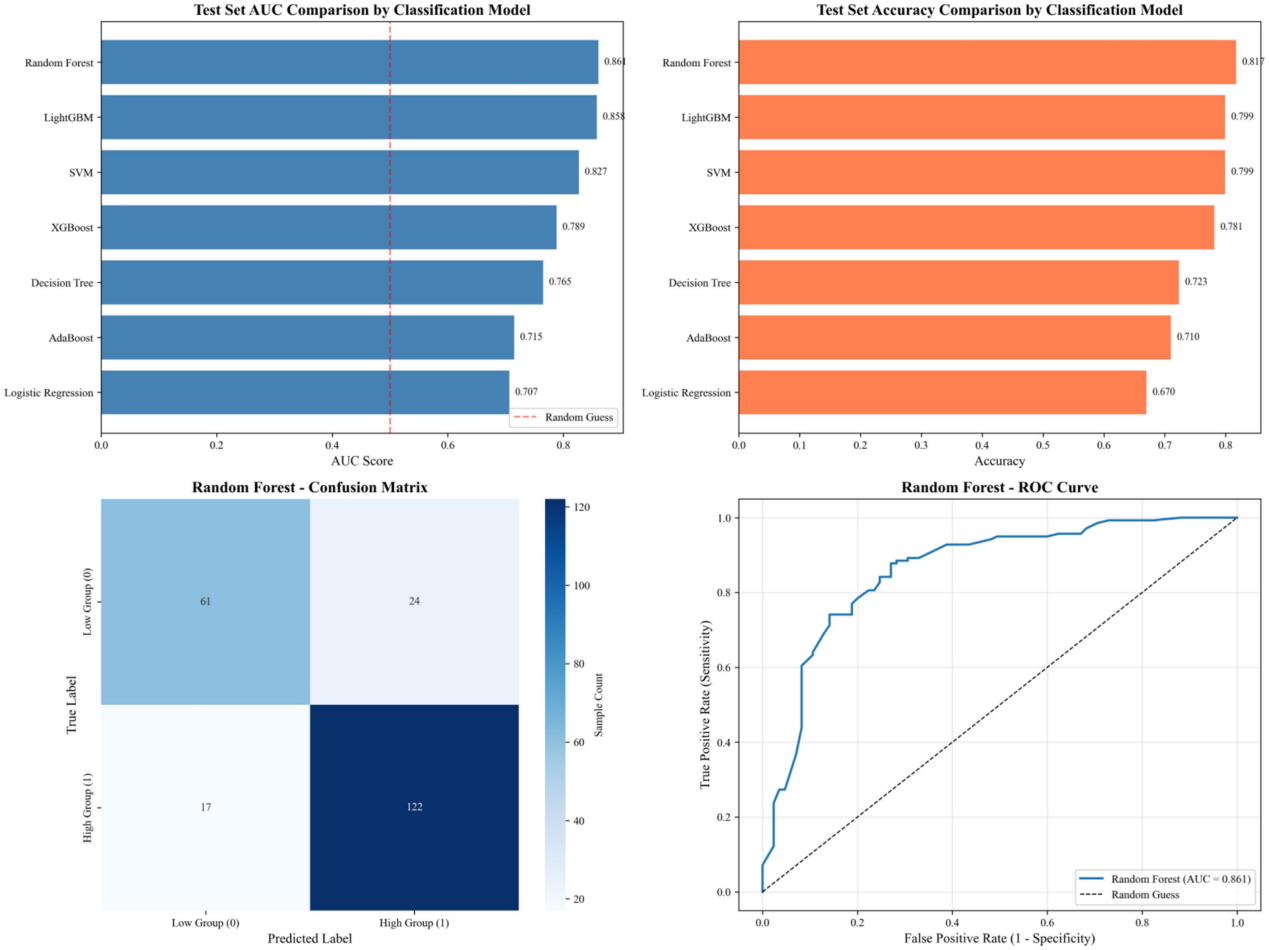
Performance evaluation of the RF model and comparison with other classification models on the test set.

Model evaluation was also performed with the application of three imbalanced data processing strategies, and the RF model was identified as the optimal one based on comprehensive evaluation including AUC and other key indicators (see Figure 2).

#### 3.2.2 Performance comparison of the ML models

##### 3.2.2.1 Precision-recall curves and decision curve analysis

**Figure 3:**
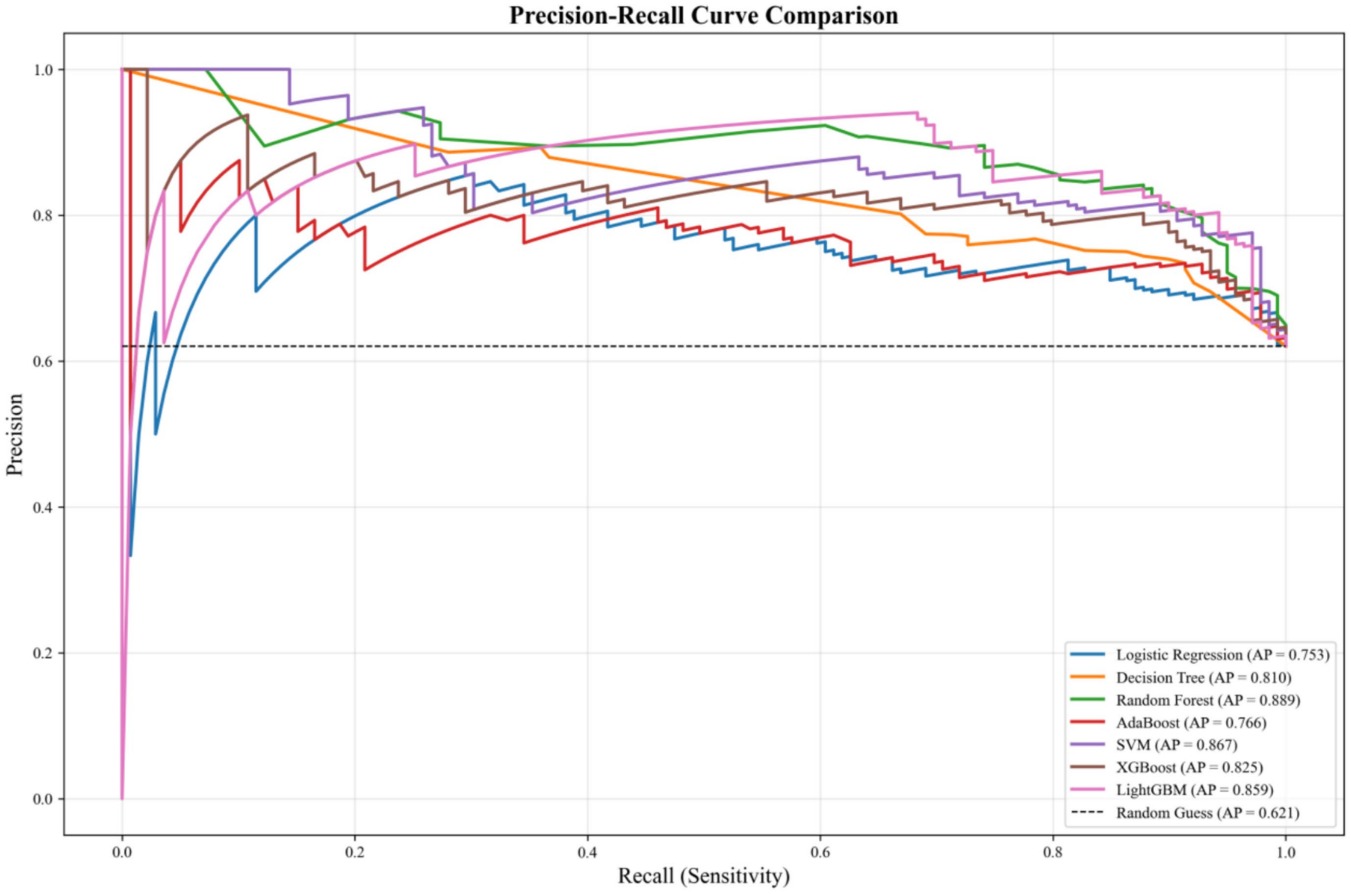
Precision–recall curves of seven ML models for predicting childcare demand.

The precision-recall curves (Figure 3) illustrate the trade-off between precision and recall for the seven ML models evaluated in this study, with the random guess baseline (AP = 0.621) shown as a dashed reference line. Among all models, RF achieved the highest average precision (AP = 0.889), followed closely by lightGBM (AP = 0.859) and XGboost (AP = 0.825), indicating superior predictive reliability and practical utility in identifying rural families with childcare demand. SVM (AP = 0.867), Adaboost (AP = 0.766), and LR (AP = 0.753) demonstrated moderate performance, while DT exhibited the lowest AP (0.810), reflecting relatively weaker predictive precision. All models consistently outperformed the random guess baseline, confirming their practical value for rural childcare policy formulation.

**Figure 4:**
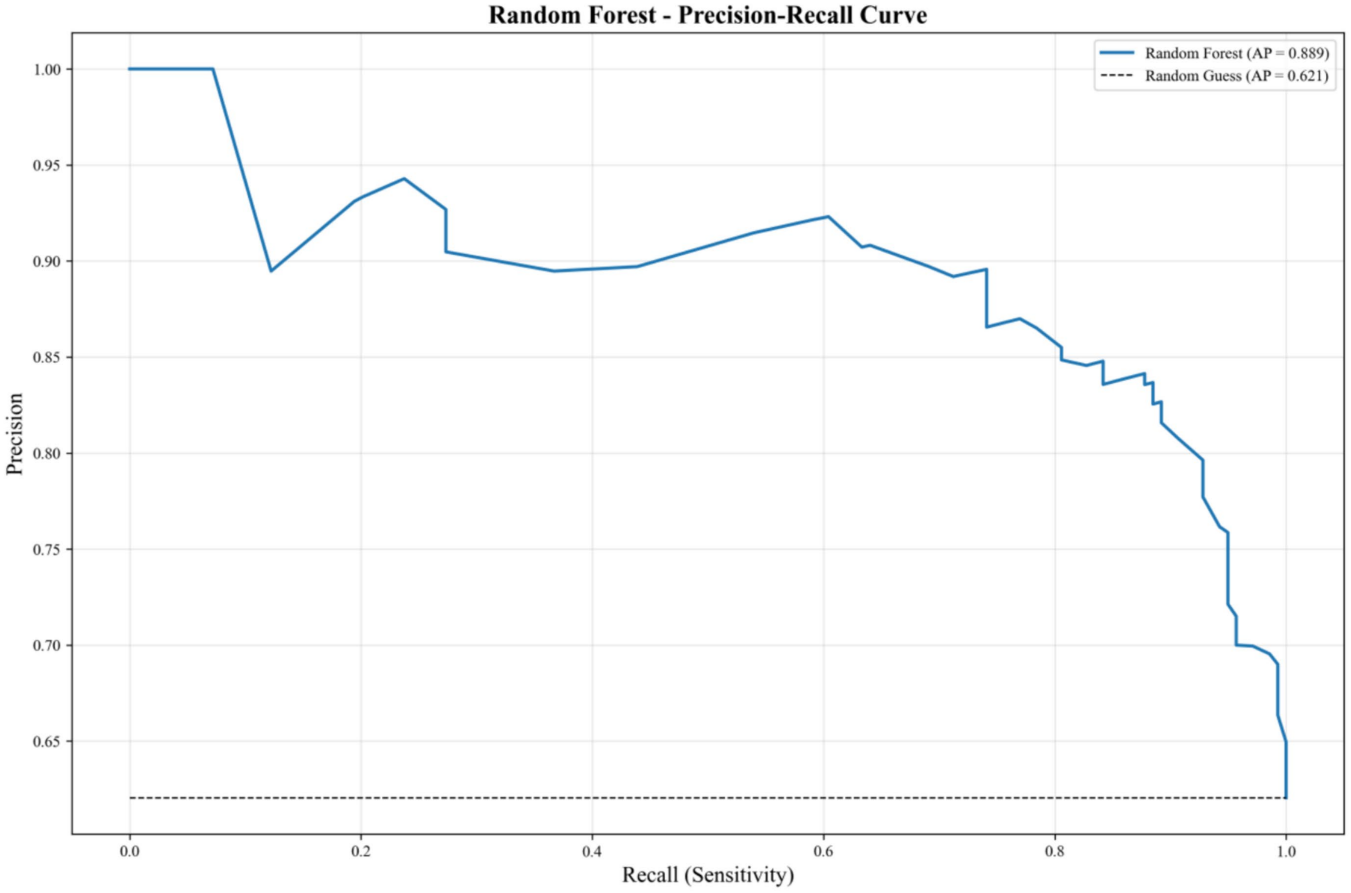
Precision–recall curve of the optimal RF model for predicting childcare demand.

To further highlight the optimal model’s performance, a dedicated precision-recall curve for RF (Figure 4) is presented. This curve shows that the RF model maintains high precision (above 0.90) across a wide range of recall values (0 to 0.6), with a gradual decline in precision as recall approaches 1.0. The model’s AP of 0.889, significantly higher than the random guess baseline (0.621), underscores its robust ability to balance precision and recall, making it the most reliable choice for identifying key factors associated with childcare demand in rural households.

**Figure 5:**
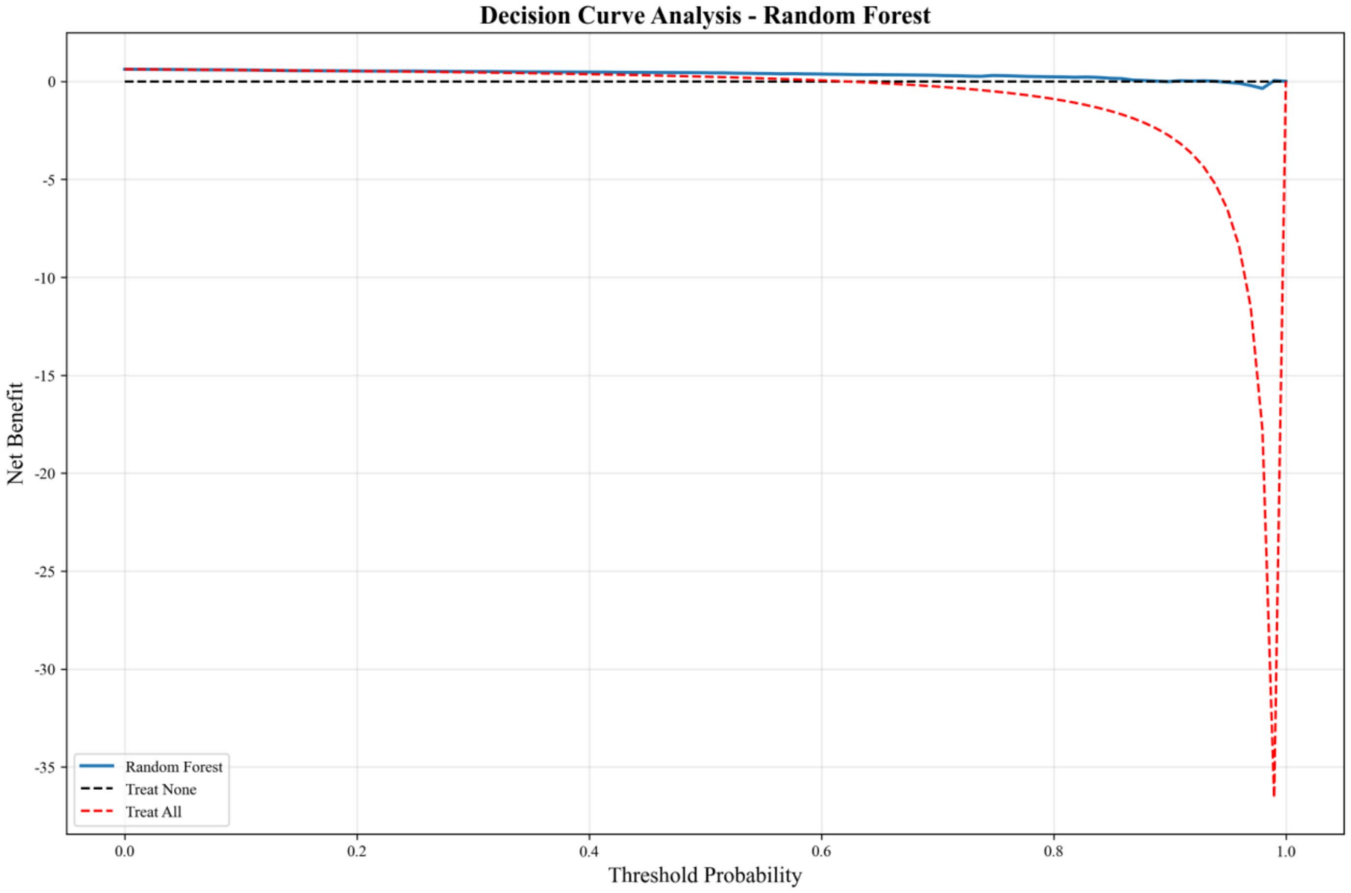
Decision curve analysis (DCA) of the optimal RF model for predicting childcare demand.

The Decision Curve Analysis (DCA) of the optimal RF model (Figure 5) quantifies its net benefit across a spectrum of threshold probabilities, in comparison to the Treat None and Treat All strategies. The model’s curve consistently lies above both reference lines across the entire relevant threshold range (0.0 ~ 0.9), confirming that the RF model not only exhibits superior statistical performance but also possesses substantial practical utility for real-world decision-making, solidifying its status as the optimal and valuable predictive tool for this study.

##### 3.2.2.2 Pairwise Mann–Whitney U-tests

**Figure 6:**
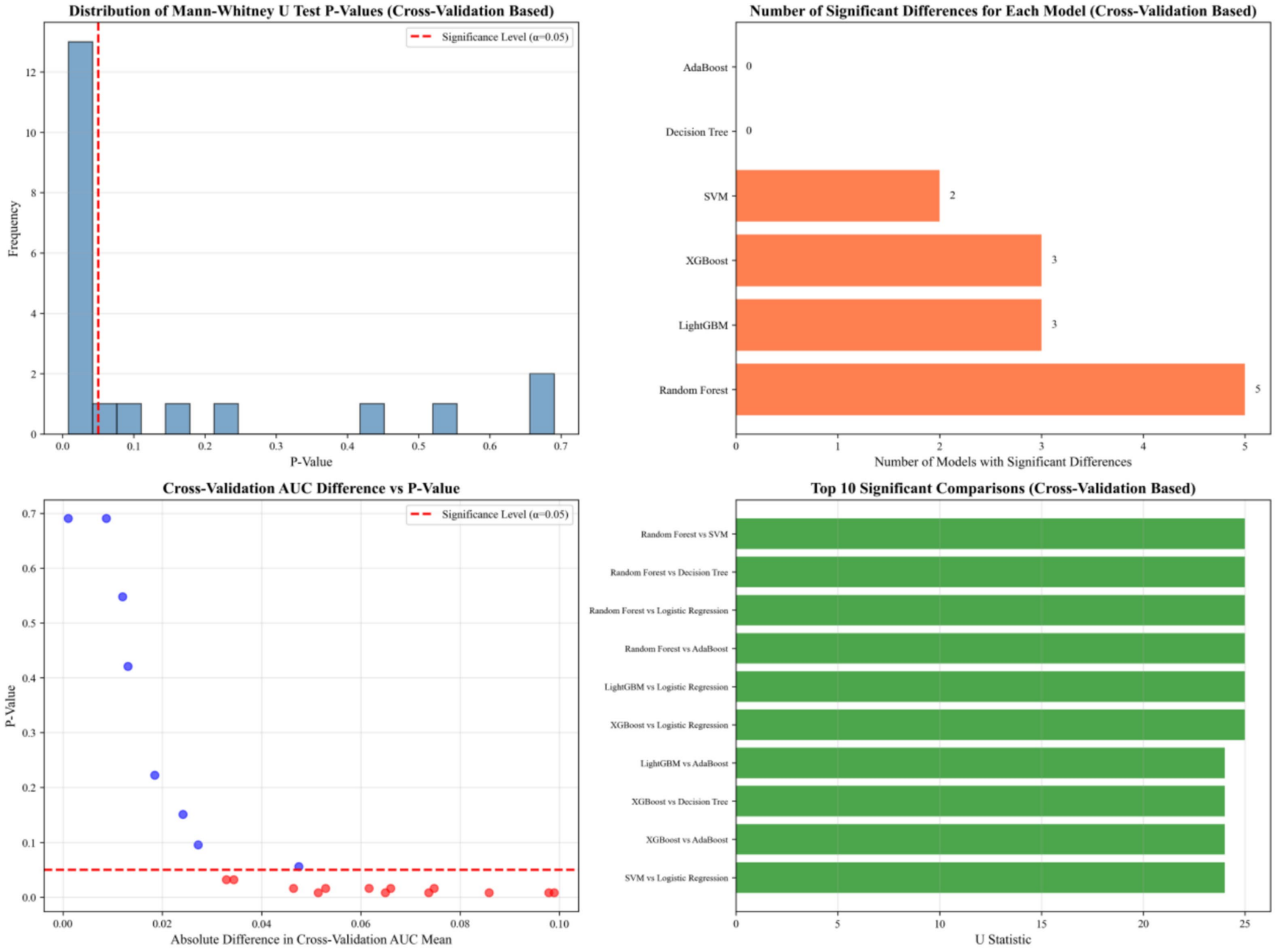
Cross-validation-based statistical comparison of model performance.

To further validate the superiority of RF, we performed pairwise Mann–Whitney U-tests to compare its AUC with that of all other models. The results are summarized in Table 5, and the corresponding cross-validation-based statistical analyses are illustrated in Figure 6.

**Table 5:** Mann–Whitney *U-*-test results for AUC comparisons between RF and other models.

Model 1	Model 2	AUC_Mean (Model 1)	AUC_Mean (Model 2)	*U* Statistics	*p*
RF	SVM	0.905	0.853	25	0.008
RF	DT	0.905	0.819	25	0.008
RF	LR	0.905	0.807	25	0.008
RF	Adaboost	0.905	0.806	25	0.008
RF	XGboost	0.905	0.872	23	0.032
RF	LightGBM	0.905	0.881	1720	0.151

As shown, RF achieved a significantly higher mean AUC (0.905) compared to SVM (0.853), DT (0.819), LR (0.807), and Adaboost (0.806), with all corresponding *p*-values being 0.008, indicating statistically significant differences. While XGboost (0.872) and lightGBM (0.881) also performed well, their mean AUCs remained lower than that of RF. The U-test results showed a *p*-value of 0.032 for the comparison between RF and XGboost, confirming a significant advantage. In contrast, the difference between RF and lightGBM was not statistically significant (*p* = 0.151), suggesting comparable discriminative performance between these two top-performing models.

These findings confirmed that the RF model outperformed most competing models in terms of predictive performance for childcare demand.

##### 3.2.2.3 Calibration curve analysis and brier score

To verify the consistency between the predicted probability of infant childcare demand and the actual observed probability, calibration curve analysis and Brier score were performed for all models. Sigmoid calibration (Platt scaling) was applied to optimize model calibration performance. The calibration curve was used to visually evaluate the agreement between predicted risks and actual outcomes, and the Brier score was used as a quantitative indicator, where lower values represent better prediction accuracy and calibration.

**Figure 7:**
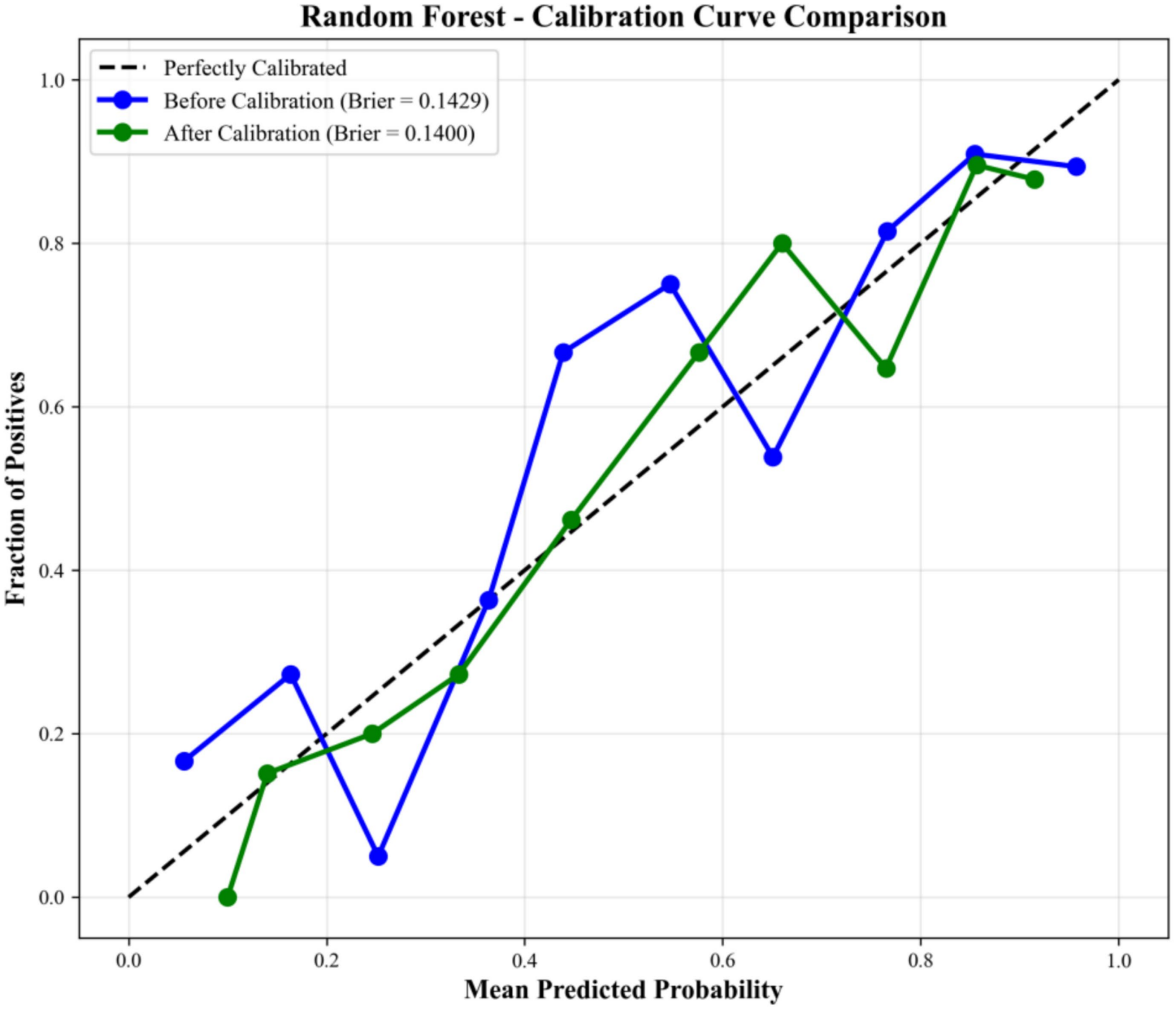
Calibration curve comparison of the RF model.

Among all models, the RF model showed the best calibration performance with the lowest Brier score (0.1429) (Figure 7), while the LR model exhibited the poorest calibration performance with the highest Brier score (0.2288). After Sigmoid calibration, the Brier score of the RF model was further improved to 0.1400, with its calibration curve closely approaching the ideal diagonal line. These results confirmed that the RF model not only had excellent discriminative ability but also maintained high consistency between predicted probabilities and actual risks, representing reliable and stable performance in predicting infant childcare demand.

### 3.3 SHAP analysis for key feature identification and model interpretation

**Figure 8:**
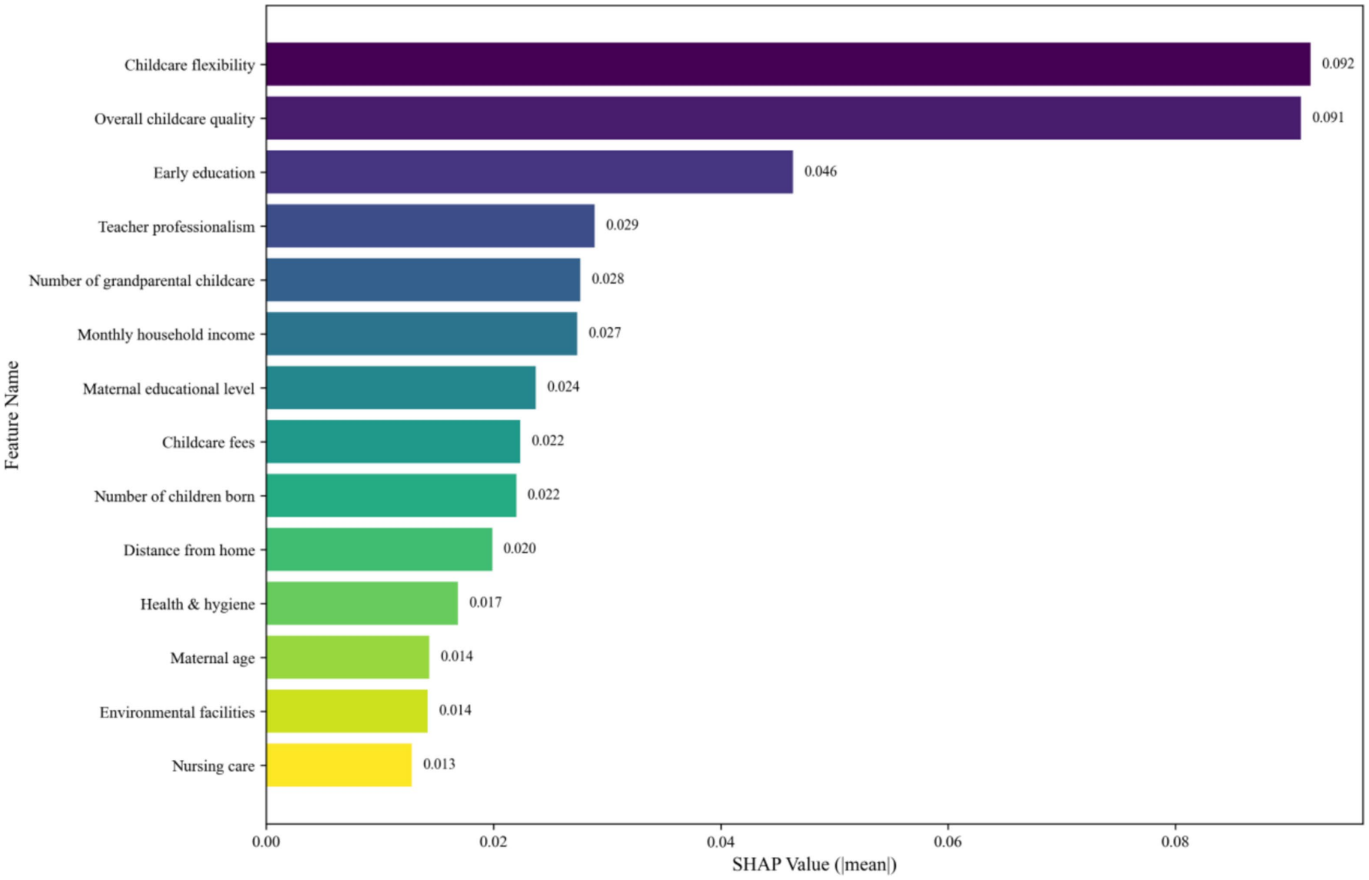
SHAP Importance of each feature for the RF model.

**Figure 9:**
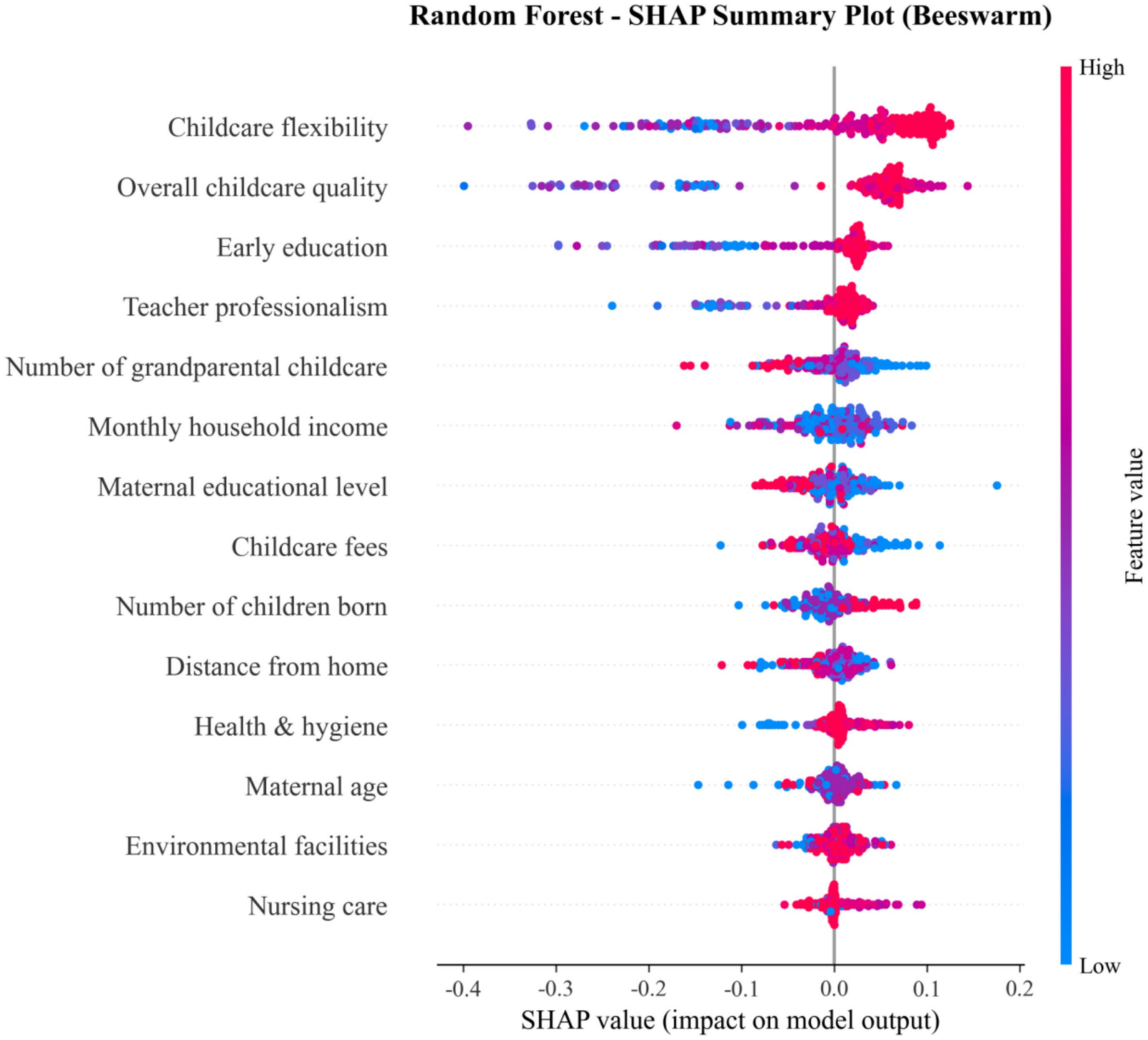
SHAP summary plot (beeswarm) for the RF model.

To interpret the black-box nature of the RF model and identify the key determinants of childcare demand, we quantified and ranked the importance of predictor variables using SHAP values (Figures 8, 9).

The SHAP importance bar plot (Figure 8) quantifies the absolute mean SHAP value for each feature, providing a clear ranking of their overall predictive power. This analysis reveals that childcare flexibility (SHAP value: 0.092) and overall childcare quality (SHAP value: 0.091) are the two most influential features, followed by early education (0.046). This ranking confirms that childcare-related factors, particularly flexibility and quality, are the primary determinants of the model’s predictions.

The SHAP summary plot (Figure 9) further visualizes both the magnitude and direction of each feature’s impact on the model output. Consistent with the importance ranking, childcare flexibility and overall childcare quality exhibit the strongest effects, with their high values (red points) strongly pushing the model predictions towards higher outcomes (positive SHAP values). Other top features, such as early education and teacher professionalism, also show a predominantly positive impact when their values are high. Conversely, features like the number of grandparental childcare display a more nuanced relationship, with both high and low values influencing predictions in different directions.

### 3.4 Threshold analysis for the optimal model and top key features

**Figure 10:**
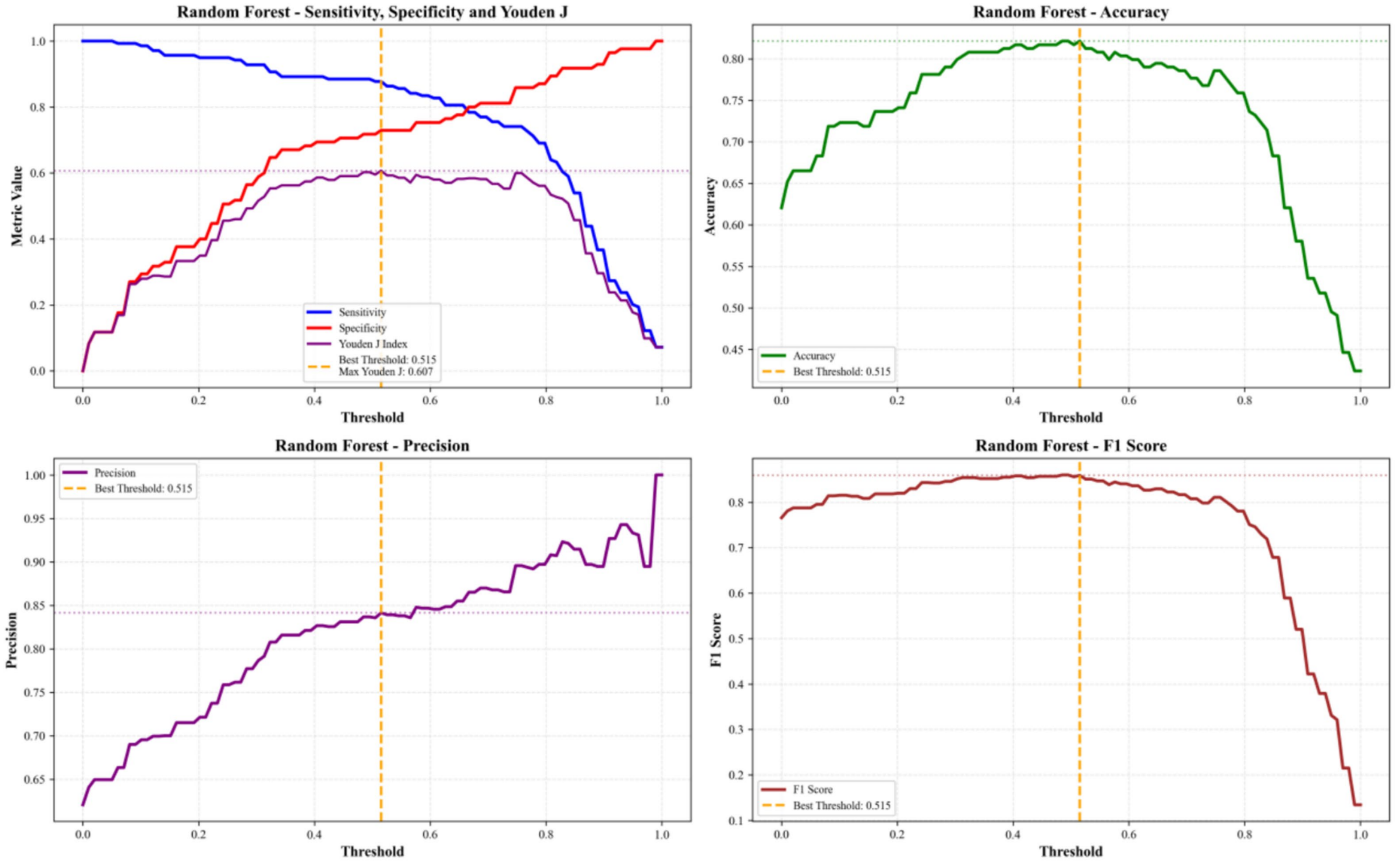
Threshold analysis of key performance metrics for the optimal RF model.

**Figure 11:**
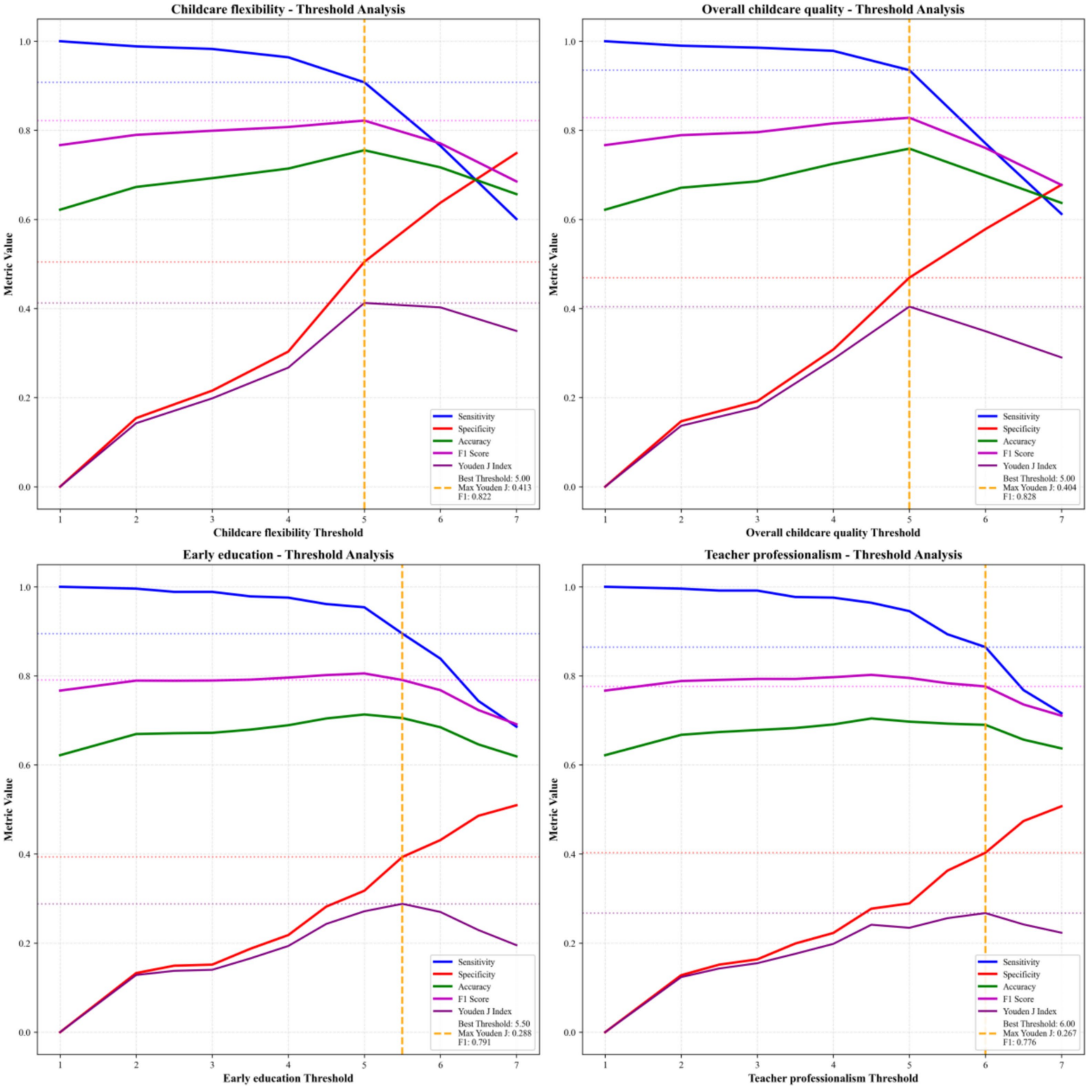
Threshold analysis of four key predictive indicators for childcare demand.

To further evaluate the practical utility of our predictive framework, we conducted threshold analyses for both the optimal RF model and its top four most important features (Figures 10, 11).

For the optimal RF model, the best classification threshold was determined to be 0.515, yielding a balanced performance with a sensitivity of 0.878, specificity of 0.729, accuracy of 0.821, precision of 0.841, F1 score of 0.859, and a Youden J index of 0.607. This threshold effectively balances the model’s ability to correctly identify positive cases while minimizing false positives, making it suitable for real-world application.

When analyzing the top four features individually, we found that childcare flexibility and overall childcare quality exhibited the strongest predictive performance at their optimal thresholds (both set at 5.0). Childcare flexibility achieved a sensitivity of 0.908, specificity of 0.505, and F1 score of 0.822, while overall childcare quality showed a slightly higher sensitivity (0.935) but lower specificity (0.469), with an F1 score of 0.828. In comparison, early education (best threshold = 5.5, F1 = 0.791) and teacher professionalism (best threshold = 6.0, F1 = 0.776) demonstrated lower overall predictive power, consistent with their lower SHAP importance rankings.

### 3.5 Multicollinearity diagnosis and correlation heatmap

**Figure 12:**
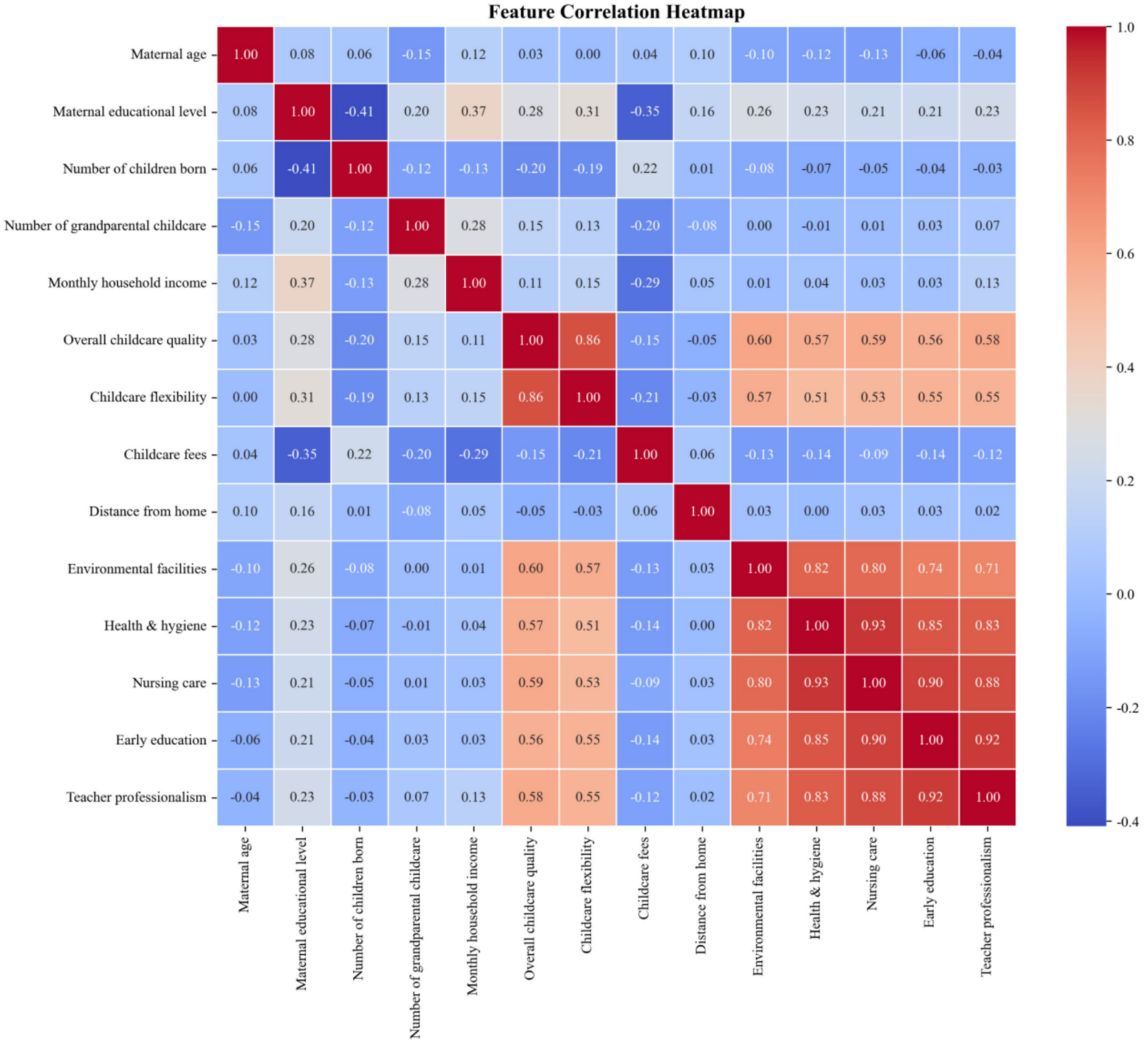
Correlation heatmap of all predictive variables.

To assess the stability and reliability of predictive modeling, multicollinearity diagnosis and correlation analysis were performed among all candidate predictive variables. A correlation heatmap (Figure 12) was constructed to visually display the pairwise correlation coefficients between variables, and the variance inflation factor (VIF) was calculated to quantitatively assess the severity of multicollinearity. Generally, a VIF value greater than 10 indicates the presence of significant multicollinearity that may bias model estimation (22).

The results of VIF analysis showed that Nursing care had a VIF value of 12.69, exceeding the threshold of 10, suggesting severe multicollinearity with other variables. Other variables had VIF values below 10, indicating no remarkable multicollinearity. The correlation heatmap further confirmed the high inter-correlation among Nursing care, Health and hygiene, Early education, and Teacher professionalism. These findings provided a reliable basis for variable screening and model construction, helping to reduce the adverse effects of multicollinearity on the performance of machine learning models.

## 4 Discussion

### 4.1 Main findings of current study

This study aimed to identify the determinants of childcare demand among rural families with infants and toddlers under 3 years old in less-developed western China, using a machine learning approach. Given the imbalanced class distribution in the dataset, three imbalance processing strategies (SMOTE, ADASYN, and RandomUnderSampler) were applied to improve model reliability. Seven machine learning algorithms were employed to construct predictive models, and the results indicated that the RF model achieved the best overall performance and was determined as the optimal model.

SHAP value analysis revealed the top four influential factors in descending order of importance: childcare flexibility, overall childcare quality, early education, and teacher professionalism. All these variables were found to exert a positive predictive effect on childcare demand. Threshold analysis further provided clear and practically meaningful cutoff values for policy design and service improvement. Specifically, the optimal thresholds were identified as 5.0 for childcare flexibility, 5.0 for overall childcare quality, 5.5 for early education, and 6.0 for teacher professionalism. These thresholds represent the minimum levels at which each factor begins to significantly drive childcare demand, offering direct operational guidance for rural childcare institutions to prioritize resource allocation, service standardization, and quality improvement. Meeting or exceeding these thresholds can effectively enhance parental willingness to use formal childcare services, thereby boosting actual demand in real-world settings. Subsequent threshold analysis further validated the practical utility of our predictive framework. For the optimal RF model, a classification threshold was identified, which balanced sensitivity and specificity, yielding robust performance metrics suitable for real-world application. Among the top four key features, childcare flexibility and overall childcare quality exhibited the strongest predictive performance. Early education and teacher professionalism showed slightly lower predictive power, which aligns with their relative importance rankings from the SHAP analysis. These findings not only confirm the model’s practical value but also provide actionable threshold values for key indicators, offering empirical guidance for the design and implementation of rural childcare policies.

Notably, the near-perfect training performance is typical for tree-based ensemble models such as random forest when fitted to high-dimensional data (22). This apparent near-perfect fitting in the training phase did not compromise generalization, as the model performed stably and consistently on the independent test set with reasonable declines in all metrics. Such a pattern is common in machine learning and does not threaten the validity of the final model, since evaluation and application rely entirely on the unseen test set rather than the training data.

### 4.2 Methodological strengths and innovations

The methodological design of this study represents a deliberate effort to address the limitations of traditional research on childcare demand and enhance the validity and depth of the findings. The selection of seven machine learning algorithms allowed for a comprehensive comparison of predictive performance. Unlike conventional statistical methods that assume linear relationships and independence among variables, machine learning models excel at capturing non-linear patterns and complex interactions in high-dimensional data (23, 33). This is particularly relevant for childcare demand, which is shaped by a multitude of interrelated factors spanning maternal, family, and service dimensions.

The use of stratified sampling in dataset partitioning ensured balanced representation of No Demand and Demand groups in both the training and test sets, mitigating the risk of bias associated with imbalanced data. Hyperparameter optimization via 5-fold cross-validation combined with grid search further enhanced the generalization ability of the models, reducing overfitting and ensuring robust performance on unseen data (14). The comprehensive evaluation metrics, including AUC, sensitivity, specificity, accuracy, precision, and F1-score, provided a holistic assessment of model performance, going beyond single metrics that may mask weaknesses in discriminative power for imbalanced outcomes. Furthermore, the use of decision curve analysis, precision-recall analysis, and Mann–Whitney *U*-test further confirmed the reliability, stability, and superior discriminative power of the random forest model, consistent with the viewpoints of Fokkema et al. (22) and Gasparini et al. (38) and further supporting that random forest is a powerful algorithm.

Notably, the application of SHAP values addressed the “black box” problem inherent in many machine learning models (24, 25, 39). By quantifying the contribution of each feature to individual predictions, SHAP analysis enabled interpretable insights into the magnitude and direction of factor influences (8). This methodological choice is critical for translating predictive models into actionable policy recommendations, as it clarifies which factors matter.

Furthermore, the cross-sectional survey design, focusing on rural childcare needs and conducted in the southwestern area of Guizhou Province, a representative less-developed rural region with a large rural population and a high proportion of migrant laborers, ensured strong ecological validity for studying the real-world situation of rural childcare demand. The purposive and multi-stage sampling strategy, which targeted eight counties (cities) and randomly selected administrative villages within each, further enhanced the representativeness of the sample for investigating rural childcare needs. Restricting questionnaire completion to mothers, the primary caregivers in rural Guizhou households, minimized response bias and ensured that data directly reflected the perceptions and decisions of key stakeholders (12).

Multicollinearity diagnostics showed that most variables had VIF values below 10, except for nursing care, which exceeded the threshold. Since tree-based models are robust to multicollinearity, we retained all variables without further variable elimination or sensitivity analysis. Unlike regression-based methods, random forest does not require strict independence between predictors and is less affected by highly correlated features (22, 38). Therefore, the collinearity detected in this study did not bias feature importance ranking or predictive performance, supporting the stability of the final model.

### 4.3 Consistency with and extensions of existing literature

The findings of this study align with and extend existing literature on childcare demand. Consistent with previous research (4, 12), family structure factors such as the number of children born significantly influence childcare demand, with multi-child families exhibiting stronger demand. This confirms that the relaxation of fertility policies in China has increased the childcare burden on rural families, thereby raising their demand for formal childcare services. Additionally, the negative association between grandparental childcare support and formal childcare demand is consistent with the conclusions of the previous studies (4, 40), highlighting the enduring role of intergenerational care in rural China’s childcare model.

These results are fully consistent with Andersen’s Behavioral Model, in which intra-family enabling resources directly shape the need for and utilization of external services. Stronger family care resources reduce reliance on formal childcare, while higher care needs increase demand, supporting the core logic of the model. Rural families with multiple children have a relatively strong demand for childcare services, whereas the demand is lower among rural families with more grandparental support.

However, this study identifies several distinct patterns compared to urban-focused research. Maternal education and household income, which are critical determinants in urban areas, show weaker influence in rural regions. This discrepancy may arise from the relatively low and homogeneous socioeconomic status of rural families, which limits the discriminatory power of such variables. Instead, childcare flexibility, overall service quality, early education content, and teacher professionalism emerged as more influential factors, which differs from urban studies that prioritize cost and accessibility (14). From the perspective of Andersen’s model, this difference reflects the stronger role of external social resources (service flexibility, quality, early education, teacher professionalism) in enabling childcare demand in rural contexts, whereas in urban areas, economic enabling resources (income, cost) play a more decisive role.

### 4.4 Key determinants of rural childcare demand and underlying mechanisms

Notably, childcare flexibility represents the most critical factor for rural families. Rural parents engaged in farming or informal labor often have irregular and seasonal working hours, making fixed full-day services poorly matched with their daily routines. Flexible arrangements such as adjustable drop-off and pick-up times, part-time or hourly care, therefore become a top priority. Scattered rural residences and limited public transport further strengthen the importance of flexible services (17). Overall childcare quality also stands out as a key concern. High-quality childcare supports children’s language, cognitive, emotional, and social development (41). In resource-constrained rural areas, families increasingly recognize the long-term benefits of supportive and developmentally appropriate care (42). This further verifies the impact of external social resource factors in the Andersen model on the childcare needs of rural families. Providing high-quality and flexible childcare services can effectively increase the childcare demand of rural families.

Accordingly, early education has become highly valued. Driven by the spread of early childhood development knowledge and urban parenting culture, rural parents are no longer satisfied with basic supervision-only care. Instead, they strongly prefer services that integrate care and early education (2), reflecting a profound shift in rural childcare attitudes. Meanwhile, teacher professionalism serves as a core component of quality childcare. Professional caregivers with adequate training can better implement age-appropriate early education activities, ensure safe and nurturing interactions, and promote healthy child development. The emphasis on teacher professionalism further demonstrates that rural families prioritize sustainable, high-quality provision over low-cost or merely accessible services.

### 4.5 Practical and policy implications of current study

The findings of this study provide meaningful implications for optimizing rural childcare services and formulating targeted policies.

First, improving childcare flexibility should be prioritized. Rural childcare institutions should diversify service models and provide flexible arrangements such as holiday care, hourly care, and seasonal care to adapt to the irregular working schedules of rural parents. Access to flexible childcare significantly increases the likelihood and working hours of rural mothers engaging in non-agricultural employment, especially given the common nonstandard working hours in rural service occupations.

Second, enhancing overall childcare quality and early education is essential. Institutions should improve facilities, hygiene standards, and daily care practices, while developing age-appropriate programs to promote children’s cognitive, social, and emotional development.

Third, strengthening the professional competence of childcare teachers should be emphasized. Targeted pre-service and in-service training should be implemented to improve teachers’ professional knowledge, practical care skills, and early education capabilities. High-quality teaching staff is crucial to ensuring service quality and promoting the healthy development of children, which in turn enhances parental recognition and demand for formal childcare services.

## 5 Strengths, limitations and future directions

This study makes several notable contributions to the existing literature. First, it fills the research gap regarding rural childcare demand in less-developed rural China. Most previous studies have focused on urban areas, while rural areas, which account for a large proportion of the population, have been overlooked. This study provides the first comprehensive analysis of rural childcare demand using a ML approach, offering unique insights into the specific needs of rural families. Second, the application of machine learning algorithms enhances the accuracy and reliability of the research. Compared to traditional statistical methods, machine learning models (especially the RF model) enable a more nuanced understanding of the determinants of childcare demand. Third, this study expands the analytical framework of childcare demand determinants. Previous rural-focused research has primarily emphasized socioeconomic and family structure factors, while this study incorporates service quality attributes and subjective maternal perceptions as core variables. Additionally, the comparison of seven machine learning algorithms and the validation of the RF model’s superiority provide strong evidence for the reliability of the results.

Despite its contributions, this study has several limitations. First, the sample was limited to southwestern area of Guizhou Province, the findings may not be fully generalizable to rural areas in other regions of China with different economic and cultural contexts. Second, the cross-sectional design of the study prevents the establishment of causal relationships between variables. For example, while we found a positive association between overall childcare quality and childcare demand, it is also possible that families with stronger demand are more likely to perceive services as high-quality. Third, owing to the constraints of research design and data availability, this study did not conduct external validation using independent datasets. The generalizability and stability of the random forest model and the identified key thresholds may therefore be weakened when applied to new samples or different regions. Fourth, this study centers on objective factors without fully exploring subjective psychological factors, including parents’ understanding of and trust in childcare institutions. Fifth, the measurement of childcare service quality relied on parental self-reports, which may be subject to response bias.

Several directions can be pursued in future studies to advance understanding of rural childcare demand. First, longitudinal studies should be conducted to establish causal relationships between service characteristics, family factors, and childcare demand, rather than relying on cross-sectional associations. Second, future research could expand the sample to other less-developed rural regions in western and central China to improve the generalizability of findings. Third, qualitative methods such as in-depth interviews and case studies can be integrated to explore subjective psychological factors, including parental trust, perceived value, and cultural attitudes toward formal childcare. Fourth, future studies may incorporate real-world operational data from rural childcare centers to validate the threshold values identified in this study and support evidence-based service design. Finally, comparative studies between rural and urban areas using the same machine learning framework could further clarify contextual differences in the drivers of childcare demand and support more targeted policy-making.

## 6 Conclusion

This study explored the determinants of rural childcare demand for infants and toddlers in western China by comparing the performance of seven machine learning algorithms, and confirmed the random forest model as the optimal predictive model for this research context. SHAP analysis identified childcare flexibility, overall childcare quality, early education, and teacher professionalism as the four core positive determinants of rural childcare demand. Threshold analysis further defined the critical values of these key factors for predicting childcare demand. Collectively, the findings of this study clarify the core influencing factors and their practical critical thresholds for rural childcare demand, providing targeted empirical insights for the optimization of rural childcare service systems and the formulation of demand-oriented childcare policies in less-developed regions.

## Data Availability

The raw data supporting the conclusions of this article will be made available by the authors, without undue reservation.
